# Adults with early-onset type 2 diabetes (aged 18–39 years) are severely underrepresented in diabetes clinical research trials

**DOI:** 10.1007/s00125-020-05174-9

**Published:** 2020-06-02

**Authors:** Jack A. Sargeant, Emer M. Brady, Francesco Zaccardi, Frances Tippins, David R. Webb, Vanita R. Aroda, Edward W. Gregg, Kamlesh Khunti, Melanie J. Davies

**Affiliations:** 1 Diabetes Research Centre, University of Leicester, Leicester General Hospital, Gwendolen Road, Leicester, LE5 4PW UK; 2 NIHR Leicester Biomedical Research Centre, University Hospitals of Leicester NHS Trust and the University of Leicester, Leicester, UK; 3 Department of Cardiovascular Sciences, University of Leicester, Leicester, UK; 4 Real-World Evidence Unit, University of Leicester, Leicester, UK; 5 Brigham and Women’s Hospital, Harvard Medical School, Boston, MA USA; 6 Department of Epidemiology and Biostatistics, School of Public Health, Imperial College London, London, UK; 7 NIHR Applied Research Collaboration East Midlands, Leicester, UK

**Keywords:** Clinical research, Clinical trials, Early-onset type 2 diabetes, Representation

## Abstract

**Aims/hypothesis:**

Early-onset adult type 2 diabetes (diagnosed between ages 18 and 39 years) is increasingly prevalent and associated with poor long-term outcomes. We hypothesised that individuals with early-onset adult type 2 diabetes were underrepresented in the prominent research trials that underpin type 2 diabetes management guidelines.

**Methods:**

We reviewed the mean age of the study populations recruited to 90 prominent trials in type 2 diabetes, including 37 cardio-renal outcomes trials across a range of pharmacological, non-pharmacological and multifactorial interventions, 28 trials from the phase III programmes of three representative glucose-lowering therapies used routinely in clinical practice (empagliflozin, liraglutide and sitagliptin) and 25 prominent trials of diabetes self-management education and support or intensive lifestyle interventions (diet or supervised exercise training). We then estimated the number of individuals within these trials who were aged between 18 and 39 years.

**Results:**

Across all 90 trials, the mean age of 268,978 participants was 63 years (range 51–69 years in individual trials). In 73 trials (81%), <5% of participants were estimated to be aged 18–39 years, despite this age group representing ~15–20% of the adult type 2 diabetes population. Twenty-nine of these trials (32%; total 164,953 participants) excluded individuals below 40 years of age altogether.

**Conclusions/interpretation:**

Guidelines for early-onset adult type 2 diabetes are extrapolated predominantly from evidence in older individuals. Strategies to support the participation of individuals with early-onset adult type 2 diabetes in future research are imperative to ensure guidelines for these high-risk individuals are evidence-based.

**Electronic supplementary material:**

The online version of this article (10.1007/s00125-020-05174-9) contains peer-reviewed but unedited supplementary material, which is available to authorised users.

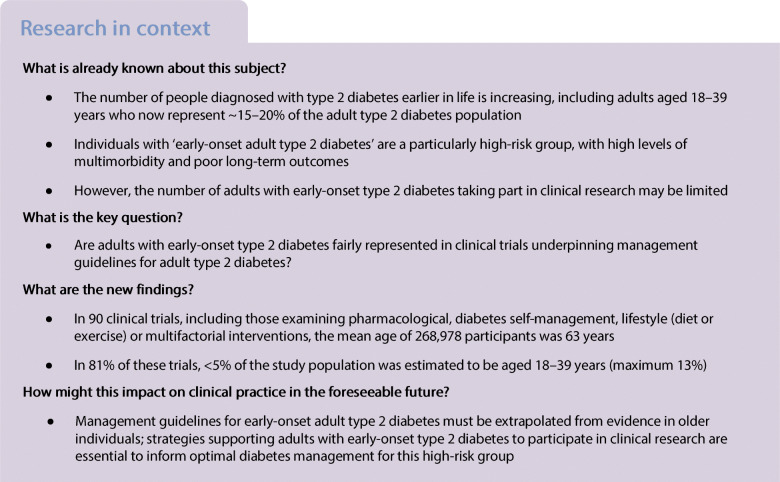



## Introduction

The prevalence of type 2 diabetes, traditionally considered a condition of mid-to-late adulthood, is increasing in younger adults (aged 18–39 years, inclusive) [1]. It is estimated that these individuals with ‘early-onset adult type 2 diabetes’ now represent up to 15–20% of the adult type 2 diabetes population worldwide [1–3]. Early-onset adult type 2 diabetes is underpinned by an extreme risk phenotype, early exposure to chronic hyperglycaemia and suboptimal self-care practices [4–6]; its impact is severe, leading to devastating micro- and macrovascular complications [1, 5, 7, 8]. Psychosocial complications including anxiety, depression and diabetes-related distress are also highly prevalent [9, 10]. Moreover, whilst mortality rates in type 2 diabetes are generally in decline, those in early-onset adult type 2 diabetes may be being left behind [11].

Ke and colleagues [12] recently highlighted that the mean age at type 2 diabetes diagnosis of individuals enrolled in 19 cardiovascular or renal (‘cardio-renal’) outcomes trials of glucose-lowering therapies (GLTs) was approximately 50–60 years. This is concerning because trials such as these heavily inform management guidelines and these guidelines are not age specific [13]. Consequently, guidelines for the use of these therapies in early-onset adult type 2 diabetes must be extrapolated from evidence in older individuals. We hypothesised that this underrepresentation of individuals with early-onset adult type 2 diabetes was not unique to these 19 trials but extended to the wider evidence base underpinning management guidelines.

## Methods

This short communication presents analyses that both complement and extend those presented by Ke and colleagues [12]. First, we reviewed the baseline age (i.e. age at enrolment) of participants in 37 cardio-renal outcomes trials in type 2 diabetes, across a range of pharmacological, non-pharmacological and multifactorial interventions. Our focus on age at enrolment represents a subtle but important, complementary difference from the analyses of Ke and colleagues, as individuals aged 18–39 years often have distinct sociocultural circumstances that may contribute to their lower representation in clinical research (e.g. family planning, early careers, new independent living). Analyses of age at diagnosis (including those by Ke and colleagues) provide valuable insight into the representation of early-onset adult type 2 diabetes as a disease phenotype within trial populations (i.e. inclusion of people diagnosed between the ages of 18 and 39 years), but nonetheless many of these participants may be older at the point of enrolment (e.g. a 50-year-old individual with disease duration of 15 years).

Second, our review extends to studies beyond just cardio-renal outcomes trials, by incorporating a representative sample of trials that underpin other fundamental components of adult type 2 diabetes management [13], including: (1) trials within the phase III research programmes of three GLTs used routinely in clinical practice and which are representative of modern phase III research programmes (the sodium–glucose co-transporter 2 inhibitor empagliflozin, the glucagon-like peptide-1 receptor agonist liraglutide, and the dipeptidyl peptidase-4 inhibitor sitagliptin); (2) prominent trials examining the efficacy of diabetes self-management education and support (DSMES) and intensive lifestyle interventions (diet or supervised exercise) in adults with type 2 diabetes.

Third, where possible, we complement our review of baseline age by estimating the number of individuals aged 18–39 years within the trials included. In manuscripts that reported the age of the recruited population as a mean (thus allowing the assumption of a normal distribution of data), we used this mean, the SD and an online calculator (http://onlinestatbook.com/2/calculators/normal_dist.html) to estimate the proportion of individuals aged 18–39 years enrolled in each trial. We then calculated this proportion of the total study sample size to estimate the number of individuals aged 18–39 years recruited.

Further details of the search strategy, study selection and data extraction are presented in electronic supplementary material (ESM) Methods.

## Results

Thirty-seven cardio-renal outcomes trials were identified, examining the effects of intensive glucose lowering towards pre-specified targets (*n* = 5), specific pharmacological GLTs (*n* = 27), and lifestyle (*n* = 1), surgical (*n* = 1) or multifactorial interventions (*n* = 3). Twenty-eight phase III trials of GLTs (empagliflozin *n* = 9, liraglutide *n* = 7, sitagliptin *n* = 12) were also included, along with 25 prominent trials of DSMES (*n* = 13) or intensive lifestyle interventions (diet *n* = 6, supervised exercise *n* = 6). Details of each trial, along with full citations, are provided in ESM Table 1. Collectively, these 90 trials recruited 268,978 individuals, with the mean age of each study population ranging from 51 to 69 years. The mean age of all trials combined, weighted for differences in sample size, was 63 years.

Twenty-three cardio-renal outcomes trials (62%; total 161,608 participants) had minimum age criteria ≥40 years, thus preventing the inclusion of individuals aged 18–39 years altogether. Nine trials had minimum age criteria of 25 (*n* = 3), 30 (*n* = 5) or 35 (*n* = 1) years, allowing the inclusion of some, but not all, individuals aged 18–39 years. Only five trials (14%) allowed the inclusion of all adults ≥18 years.

**Fig. 1:**
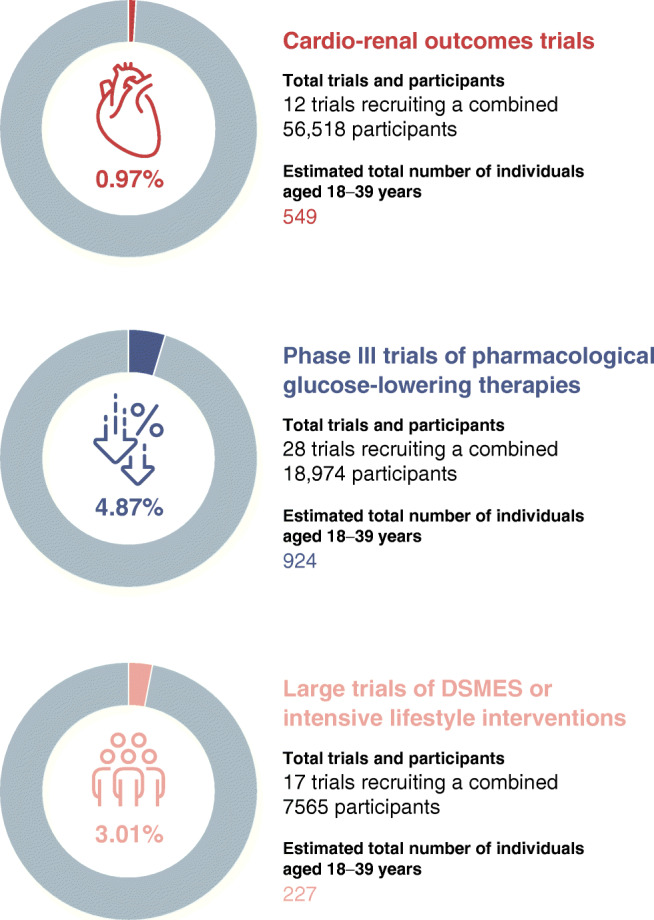
Estimated proportions of individuals aged 18–39 years participating in cardio-renal outcomes trials, phase III trials of pharmacological GLTs used routinely in clinical practice, and prominent trials exploring the efficacy of DSMES or intensive lifestyle interventions (diet or supervised exercise training) in adults with type 2 diabetes. Analyses in cardio-renal outcomes and DSMES or intensive lifestyle intervention trials include only trials for which at least some individuals aged 18–39 years would have been eligible (i.e. minimum age criteria <40 years). A further 23 cardio-renal outcomes trials (total 161,608 participants) and six DSMES/intensive lifestyle intervention trials (total 3345 participants) had minimum age criteria ≥40 years, thus excluding individuals aged 18–39 years altogether. Two cardio-renal outcomes trials (total 20,132 participants) and two DSMES trials (total 836 participants) were also excluded from analyses as they did not report age as mean and SD, thus preventing estimation of the proportion of individuals aged 18–39 years

In the 14 trials for which at least some individuals aged 18–39 years were eligible (i.e. minimum age criteria <40 years; total 76,650 participants), the mean age of the recruited populations ranged from 53 to 66 years, with a weighted mean age of 62 years. Twelve of these 14 trials reported age as the mean and SD, thus allowing estimation of the number of individuals aged 18–39 years (the remaining two trials reported the median with or without interquartile range) (ESM Table 1, refs 32 and 39). Collectively, <1% of the combined population in these 12 trials were estimated to fall within the ages of 18–39 years (range 0.2–4.8%) (Fig. 1). Data for each trial are presented in ESM Fig. 1.

All 28 phase III trials of GLTs (total 18,974 participants) had minimum age criteria of 18 years, except one which was 21 years. The mean age of recruited participants ranged from 51 to 68 years, with a weighted mean age of 56 years. Data stratified by GLT are provided in ESM Table 2. Results remained similar after exclusion of three trials that specifically recruited individuals with additional comorbidities (hypertension *n* = 1, renal insufficiency *n* = 2), which had notably higher mean ages (ESM Table 1, refs 61, 62 and 81). It was estimated that individuals aged 18–39 years represented <5% of the combined population (range 0.2–13.0%) (Fig. 1; ESM Fig. 2).

Six DSMES or intensive lifestyle intervention trials (24%; total 3345 participants) excluded individuals aged 18–39 years. The minimum age criteria of the remaining trials were 18 (*n* = 6), 20 (*n* = 1), 21 (*n* = 1), 25 (*n* = 1), 30 (*n* = 5), 35 (*n* = 1) and 39 (*n* = 1) years. Three trials did not report minimum age criteria, but inferred that all individuals with type 2 diabetes were eligible. In the 19 studies with minimum age criteria <40 years (total 8401 participants), the weighted mean age was 59 years (range 52–69 years). Stratified data for DSMES, dietary and supervised exercise interventions are provided in ESM Table 3.

Two studies did not report the SD, preventing estimation of the number of individuals aged 18–39 years. It was estimated that approximately 3% of the combined population in the remaining 17 trials were aged 18–39 years (range 0.01–11.2%) (Fig. 1; ESM Fig. 3).

## Discussion

Our analyses highlight that individuals with early-onset adult type 2 diabetes, aged 18–39 years, are severely underrepresented in clinical research trials. Importantly, this is not isolated to cardio-renal outcomes trials of pharmacological GLTs but extends to similar outcomes trials examining non-pharmacological and multifactorial approaches, as well as phase III trials of GLTs used routinely in clinical practice and other large trials of DSMES or intensive lifestyle interventions. Notably, our analyses include individuals who remained within the ages of 18–39 years at trial enrolment and exclude individuals who were diagnosed with early-onset adult type 2 diabetes but were ≥40 years when enrolled.

Our findings are important as they suggest that the older age of populations seen in cardio-renal outcomes trials may not solely be a result of purposive eligibility criteria designed to increase statistical power by observing higher rates of adverse cardio-renal events. Other factors, including the often complex busy lives of individuals aged 18–39 years and with potentially differing priorities from those of older adults, may preclude their involvement in clinical research even when eligibility criteria permit their enrolment. Some of these factors (including independent living, lower perceived vulnerability and less established future plans) may be similar to those identified in adolescents with type 2 diabetes (defined as age 15–19 years) [14]. Given that women aged 18–39 years are of childbearing age, family planning also remains an important factor that may preclude their involvement. It is imperative that strategies are implemented to specifically support the effective participation of individuals aged 18–39 years in clinical diabetes research trials, where safe, to ensure that management guidelines are appropriately evidence-based, particularly given that the number of individuals with early-onset adult type 2 diabetes will likely continue to rise.

These individuals also represent a particularly high-risk group with poor long-term outcomes (including greater multimorbidity, risk of complications and years of life lost [1, 5, 7, 8, 11]). Distinct management strategies or approaches may therefore be optimal for this group. For example, they may benefit from earlier, more aggressive intervention to halt the early development of severe complications, whilst novel approaches to long-term management and ongoing clinical consultation may also be effective. Sex-specific strategies may also be warranted. However, trials specifically in individuals with type 2 diabetes aged 18–39 years are required to test these hypotheses. In this regard, the inclusion of ‘interventions in the under 40s with type 2 diabetes’ in the 2018 UK NHS Research Needs Assessment and accordant research recommendations from the National Institute for Health and Care Excellence are encouraging [15, 16]. Data from studies addressing these calls are eagerly anticipated.

## Electronic supplementary material

ESM 1 (PDF 860 kb)

## Data Availability

The dataset generated during this study is available from the corresponding author on reasonable request.

## Abbreviations

DSMES: Diabetes self-management education and support
GLT: Glucose-lowering therapy
