# Supplementary material for: Adults with early-onset type 2 diabetes (aged 18–39 years) are severely underrepresented in diabetes clinical research trials
Source: Diabetologia. 2020 Jun 2;63(8):1516–20. doi: 10.1007/s00125-020-05174-9 (PMC7351852; doi:10.1007/s00125-020-05174-9)
Supplement: Supplementary file 1 — (PDF 860 kb) [file 125_2020_5174_MOESM1_ESM.pdf]

ELECTRONIC SUPPLEMENTARY MATERIAL

Adults with early-onset type 2 diabetes (aged 18-39 years) are severely underrepresented in diabetes clinical research trials

Jack A. Sargeant[1,2], Emer M. Brady[1,3], Francesco Zaccardi[1,4], Frances Tippins[1], David R. Webb[1,2], Vanita R. Aroda[5], Edward W. Gregg[6], Kamlesh Khunti[1,7], Melanie J. Davies[1,2]

[1]Diabetes Research Centre, University of Leicester, Leicester General Hospital, Gwendolen Road, Leicester LE5 4PW, UK

[2]NIHR Leicester Biomedical Research Centre, University Hospitals of Leicester NHS Trust and the University of Leicester, Leicester, UK

[3]Department of Cardiovascular Sciences, University of Leicester, Leicester, UK

[4]Real-World Evidence Unit, University of Leicester, Leicester, UK

[5]Brigham and Women's Hospital, Harvard Medical School, Boston, MA, USA

[6]Department of Epidemiology and Biostatistics, School of Public Health, Imperial College London, London, UK

[7]NIHR Applied Research Collaboration East Midlands, Leicester, UK

ORCID IDs:

Jack A. Sargeant: 0000-0003-0395-7329

Emer M. Brady: 0000-0002-4715-9145

Melanie J. Davies: 0000-0002-9987-9371

Corresponding author contact details:

Jack A. Sargeant, Diabetes Research Centre, University of Leicester, Leicester General Hospital, Gwendolen Road, Leicester, LE5 4PW, UK. E-mail: js928@leicester.ac.uk

Abbreviations

CVD – cardiovascular disease

DPP4i – dipeptidyl peptidase 4 inhibitor

DSMES – diabetes self-Management education and support

GLP-1RA – glucagon-like peptide-1 receptor agonist

GLT – glucose-lowering therapy

IQR – interquartile range

NR – not reported

SGLT2i – sodium-glucose co-transporter 2 inhibitor

TZD - thiazolidinedione

Electronic Supplementary Methods

Study Selection

Firstly, we searched for published manuscripts (from inception to 27th September 2019) reporting demographic data of the study populations recruited to cardio-renal outcomes trials in adults with type 2 diabetes. This included both ongoing (but fully recruited) and completed trials. To do this, we:

i. Searched the online database, PubMed, using search terms related to type 2 diabetes, mortality and cardio-renal outcomes.
ii. Examined the reference lists of review articles and meta-analyses identified by our search.
iii. Examined the reference list of the ADA-EASD consensus report for the management of hyperglycaemia in adults with type 2 diabetes [1].

Secondly, we identified studies contained within the Phase III research programmes for empagliflozin and liraglutide through a targeted search for trials in the "EMPA-REG" and "LEAD" programmes, respectively, and for manufacturer-funded trials examining the efficacy of sitagliptin on improving glycaemic control in isolation or alongside alternative glucose-lowering therapies (GLTs). We pre-specified trials of empagliflozin, liraglutide and sitagliptin as they:

a) Are representative of their class of therapy.
b) Are the most commonly prescribed, were the first licensed worldwide or had the earliest cardio-renal outcomes data.
c) Have Phase III research programmes that are representative of those required for modern licencing of pharmacological GLTs in type 2 diabetes.

Thirdly, prominent trials of diabetes self-management education and support (DSMES) and intensive lifestyle interventions (diet and/or structured exercise training) were identified by review of the ADA-EASD consensus report for management of hyperglycaemia in adults with type 2 diabetes [1], and subsequent examination of relevant reference lists. Specifically, we selected six DSMES trials that were highlighted within a recent narrative review as those examining the most well-established DSMES programmes in type 2 diabetes over the past 10 years [2], and supplemented these with trials recruiting more than 500 participants identified in two systematic reviews of DSMES on glycaemic control and all-cause mortality, respectively [3, 4]. One trial was excluded because it recruited participants with type 1 or type 2 diabetes [5], whilst another was excluded because it examined both DSMES and, in a subset, concurrent pharmacological therapy [6].

We selected three dietary intervention trials which were cited directly within the ADA-EASD consensus report [1], examining the impact of meal replacement therapy, a Mediterranean-style diet and the "Dietary Approaches to Stop Hypertension" (DASH) diet. We included these trials as they were specifically in type 2 diabetes and did not include cited trials that recruited participants with overweight or obesity with or without type 2 diabetes. We also selected representative trials of low-fat, low-carbohydrate, low-glycaemic index, and high-protein diets from two systematic reviews cited within the ADA-EASD consensus report [7, 8]. A large RCT of the commercially-available Weight Watchers programme was identified [9], but it did not report the age of recruited population and was thus excluded.

To explore studies of supervised exercise training, we selected a large RCT which was cited directly in the ADA-EASD consensus report [1], supplemented by a further five trials which examined the impact of exercise training in more than 100 adults with type 2 diabetes via five systematic reviews that were also cited [10–14]. The large "Early Actid" trial (more than 500

participants) was selected from a systematic review of pedometer use to support light-intensity (walking) physical activity in type 2 diabetes [15]. However, this trial examined the effect of dietary counselling with or without pedometer use and was subsequently categorised as a DSMES trial within our analyses.

Data Extraction and Analysis

We extracted data from selected manuscripts on the number of participants recruited (*n*), the age eligibility criteria, and the age of the recruited population. The latter were extracted as mean and standard deviation (SD) or median and interquartile range (IQR), as reported. SD was calculated from standard error of the mean (SE) or 95 % confidence intervals where required [16]. The pooled mean age of individuals recruited to various groups of studies (e.g. all studies collectively, cardio-renal outcomes trials alone, Phase III trials alone) were summarised as a weighted mean accounting for differences in study sample size.

Electronic Supplementary Results

ESM Table 1 – Details of all studies reviewed

First Author (year) [study ref.]	Trial Acronym	Study Category	Intervention and Comparator	Study Sample Size	Minimum Age Criterion (years)	Age of Recruited Population (years) *Mean (SD) unless otherwise specified*	Proportion Aged 18 to 39 years (%)
Completed cardiovascular and/or renal outcomes trials							
Turner (1998) [17]	UKPDS 34	Intensive glucose lowering	Intensive therapy with metformin vs conventional treatment; target fasting plasma glucose concentration of 6mmol/L	1704[a]	25	53 (8)	4.0
Turner (1998) [18]	UKPDS 33	Intensive glucose lowering	Intensive therapy with a sulphonylurea or insulin vs conventional treatment; target fasting plasma glucose concentration of 6mmol/L	3867	25	53.3 (8.6)	4.8
Duckworth (2009) [19]	VADT	Intensive glucose lowering	Intensive vs standard therapy with multiple agents according to pre-specified algorithm (stratified by BMI); target 1.5% absolute reduction in HbA1c in intensive vs standard therapy group	1791	41	60.4 (9.0)	Not eligible
Gerstein (2008) [20]	ACCORD	Intensive glucose lowering	Intensive (individualised) therapy with multiple agents vs standard therapy; target HbA1c < 6.0% (7.0 – 7.9% in standard therapy group)	10251	40	62.2 (6.8)	Not eligible
Patel (2008) [21]	ADVANCE	Intensive glucose lowering	Intensive therapy with gliclazide and other therapies where required vs standard therapy; target HbA1c ≤ 6.5% (target in standard therapy group as per local guidelines)	11140	55	66 (6)	Not eligible
Kooy (2009) [22]	HOME	Specific GLT – Metformin	Metformin vs placebo	390	30	61.3 (10.3)	1.5
Neal (2017) [23]	CANVAS / CANVAS-R	Specific GLT – SGLT2i	Canagliflozin vs placebo	10142	30	63.3 (8.3)	0.2

First Author (year) [study ref.]	Trial Acronym	Study Category	Intervention and Comparator	Study Sample Size	Minimum Age Criterion (years)	Age of Recruited Population (years) *Mean (SD) unless otherwise specified*	Proportion Aged 18 to 39 years (%)
Perkovic (2019) [24]	CREDENCE	Specific GLT – SGLT2i	Canagliflozin vs placebo	4401	30	63.0 (9.2)	0.5
Wiviott (2019) [25]	DECLARE-TIMI 58	Specific GLT – SGLT2i	Dapagliflozin vs placebo	17160	40	64.0 (6.8)	Not eligible
Zinman (2015) [26]	EMPA-REG OUTCOME	Specific GLT – SGLT2i	Empagliflozin vs placebo	7020	18	63.0 (8.7)	0.3
Gerstein (2019) [27]	REWIND	Specific GLT – GLP-1RA	Dulaglutide vs placebo	9901	50	66.2 (6.5)	Not eligible
Hernandez (2018) [28]	HARMONY	Specific GLT – GLP-1RA	Albiglutide vs placebo	9463	40	64.1 (8.7)	Not eligible
Husain (2019) [29]	PIONEER 6	Specific GLT – GLP-1RA	Semaglutide (oral) vs placebo	3183	50	66 (7)	Not eligible
Marso (2016a) [30]	SUSTAIN 6	Specific GLT – GLP-1RA	Semaglutide (subcutaneous) vs placebo	2397	50	64.6 (7.4)	Not eligible
Marso (2016b) [31]	LEADER	Specific GLT – GLP-1RA	Liraglutide vs placebo	9340	50	64.3 (7.2)	Not eligible
Holman (2017) [32]	EXSCEL	Specific GLT – GLP-1RA	Exenatide (extended release) vs placebo	14752	18	62 (56 – 68)[b]	Not estimated (normal distribution not assumed)
Pfeffer (2015) [33]	ELIXA	Specific GLT – GLP-1RA	Lixisenatide vs placebo	6068	30	60.3 (9.7)	1.4
Gantz (2017) [34]	OMNEON	Specific GLT – DPP4i	Omarigliptin vs placebo	4192	40	63.6 (8.5)	Not eligible
Green (2015) [35]	TECOS	Specific GLT – DPP4i	Sitagliptin vs placebo	14671	50	65.5 (8.0)	Not eligible

First Author (year) [study ref.]	Trial Acronym	Study Category	Intervention and Comparator	Study Sample Size	Minimum Age Criterion (years)	Age of Recruited Population (years) *Mean (SD) unless otherwise specified*	Proportion Aged 18 to 39 years (%)
Rosenstock (2019a) [36]	CARMELINA	Specific GLT – DPP4i	Linagliptin vs placebo	6979	18	65.9 (9.1)	0.2
Rosenstock (2019b) [37]	CAROLINA	Specific GLT – DPP4i	Linagliptin vs glimepiride	6033	40	64.0 (9.5)	Not eligible
Scirica (2013) [38]	SAVOR-TIMI 53	Specific GLT – DPP4i	Saxagliptin vs placebo	16492	40	65.1 (8.6)	Not eligible
White (2013) [39]	EXAMINE	Specific GLT – DPP4i	Alogliptin vs placebo	5380	18	61 (IQR NR)[b]	Not estimated (normal distribution not assumed)
Dormandy (2005) [40]	PROactive	Specific GLT – TZD	Pioglitazone vs placebo	5238	35	61.8 (7.7)	0.2
Yoshii (2017) [41]	PROFIT-J	Specific GLT – TZD	Pioglitazone vs placebo	481	55	69.0 (7.2)	Not eligible
Vaccaro (2017) [42]	TOSCA.IT	Specific GLT – TZD	Pioglitazone vs sulphonylurea	3028	50	62.3 (6.5)	Not eligible
Home (2009) [43]	RECORD	Specific GLT – TZD	Rosiglitazone vs metformin and sulphonylurea dual-therapy	4447	40	58.4 (8.3)	Not eligible
Gerstein (2012) [44]	ORIGIN	Specific GLT – Insulin	Insulin glargine vs standard care	12537	50	63.5 (7.9)	Not eligible
Marso (2017) [45]	DEVOTE	Specific GLT – Insulin	Insulin degludec vs insulin glargine	7637	50	65.0 (7.4)	Not eligible
Raz (2009) [46]	HEART2D	Specific GLT – Insulin	Postprandial insulin lispro three-times daily vs Neutral Protamine Hagedorn insulin twice daily or insulin glargine once daily	1115	30	61.0 (9.8)	1.2
Lincoff (2014) [47]	AleCardio	Other pharmacological therapy	Aleglitazar vs placebo	7226	18	61 (10)	1.4

**First Author (year) [study ref.]**	**Trial Acronym**	**Study Category**	**Intervention and Comparator**	**Study Sample Size**	**Minimum Age Criterion (years)**	**Age of Recruited Population (years)** *Mean (SD) unless otherwise specified*	**Proportion Aged 18 to 39 years (%)**
Wing (2013) [48]	Look AHEAD	Intensive lifestyle intervention	Intensive lifestyle intervention (promoting weight loss through reduced caloric intake and increased physical activity) vs diabetes support and education	5145	45	58.7 (6.9)	Not eligible
Frye (2009) [49]	BARI 2D	Surgery and pharmacological therapy approach[c]	Prompt revascularisation with intensive medical therapy vs medical therapy alone and insulin sensitisation vs insulin provision	2368	25	62.4 (8.9)	0.4
Gaede (2003) [50]	STENO-2	Multifactorial intervention	Intensive multifactorial intervention (containing stepwise implementation of behaviour modification and pharmacological therapy targeting hyperglycaemia, hypertension, dyslipidaemia and microalbuminuria, plus aspirin for secondary prevention of CVD) vs standard therapy	160	40	55.1 (7.2)	Not eligible
Hansen (2013) [51]	DCGP	Multifactorial intervention	Intensive multifactorial intervention containing structured personalised care vs standard therapy	1381	40	65.4 (55.7 – 73.6)[b]	Not estimated (normal distribution not assumed)
Ueki (2017) [52]	J-DOIT3	Multifactorial intervention	Intensive multifactorial intervention (targeting hyperglycaemia, dyslipidaemia and hypertension) vs conventional therapy	2540	45	59.0 (6.4)	Not eligible
Ongoing cardiovascular and/or renal outcomes trials							
Cannon (2018) [53]	VERTIS-CV	Specific GLT – SGLT2i	Ertugliflozin vs placebo	8238	40	64.4 (8.1)	Not eligible

First Author (year) [study ref.]	Trial Acronym	Study Category	Intervention and Comparator	Study Sample Size	Minimum Age Criterion (years)	Age of Recruited Population (years) *Mean (SD) unless otherwise specified*	Proportion Aged 18 to 39 years (%)
Representative Phase III studies of glucose-lowering therapies used routinely in clinical practice							
Roden (2013) [54]	EMPA-REG MONO	Specific GLT – SGLT2i	Empagliflozin vs placebo vs sitagliptin (all monotherapy)	899	18	55 (11)	7.3
Häring (2014) [55]	EMPA-REG MET	Specific GLT – SGLT2i	Empagliflozin vs placebo (all background metformin)	638	18	55.7 (9.9)	4.6
Häring (2013) [56]	EMPA-REG METSU	Specific GLT – SGLT2i	Empagliflozin vs placebo (all background metformin and sulphonylurea)	666	18	57.1 (9.2)	2.5
Kovacs (2014) [57]	EMPA-REG PIO	Specific GLT – SGLT2i	Empagliflozin vs placebo (all background pioglitazone with or without metformin)	498	18	54.5 (9.8)	5.7
Rosenstock (2015) [58]	EMPA-REG BASAL	Specific GLT – SGLT2i	Empagliflozin vs placebo (all background basal insulin with or without metformin and/or sulphonylurea)	494	18	58.8 (9.9)	2.3
Rosenstock (2014) [59]	EMPA-REG MDI	Specific GLT – SGLT2i	Empagliflozin vs placebo (all background multiple daily injections of insulin with or without metformin)	563	18	56.7 (9.5)	3.1
Ridderstråle (2014) [60]	EMPA-REG H2H SU	Specific GLT – SGLT2i	Empagliflozin vs glimepiride (all background metformin)	1545	18	56.0 (10.4)	5.1
Tikkanen (2015) [61]	EMPA-REG BP	Specific GLT – SGLT2i	Empagliflozin vs placebo (various, but stable, background GLTs but all undergoing pharmacological treatment for hypertension)	823	18	62 (9)	0.5
Barnett (2014) [62]	EMPA-REG RENAL	Specific GLT – SGLT2i	Empagliflozin vs placebo (various, but stable, background GLTs, excluding SGLT2is, but all with eGFR ≥ 15 and < 90 ml/min per $1.73m^2$)	290	18	62.6 (8.3)	0.2

First Author (year) [study ref.]	Trial Acronym	Study Category	Intervention and Comparator	Study Sample Size	Minimum Age Criterion (years)	Age of Recruited Population (years) *Mean (SD) unless otherwise specified*	Proportion Aged 18 to 39 years (%)
Marre (2009) [63]	LEAD 1	Specific GLT – GLP-1RA	Liraglutide (in combination with glimepiride with or without rosiglitazone) vs placebo (plus glimepiride with or without rosiglitazone)	1041	18	56 (10)	4.5
Nauck (2009) [64]	LEAD 2	Specific GLT – GLP-1RA	Liraglutide vs placebo vs glimepiride (all in combination with metformin)	1091	18	57 (9)	2.3
Garber (2009) [65]	LEAD 3	Specific GLT – GLP-1RA	Liraglutide vs glimepiride (all on various background monotherapy; excluding insulin)	746	18	53.0 (10.8)	9.7
Zinman (2009) [66]	LEAD 4	Specific GLT – GLP-1RA	Liraglutide vs placebo (all in combination with metformin and rosiglitazone)	533	18	55 (10)	5.5
Buse (2009) [67]	LEAD 5	Specific GLT – GLP-1RA	Liraglutide vs exenatide (all on background maximal tolerated doses of metformin, sulphonylurea or both)	464	18	56.7 (10.3)	4.3
Russell-Jones (2009) [68]	LEAD 6	Specific GLT – GLP-1RA	Liraglutide vs placebo vs insulin glargine (all in combination with metformin and glimepiride and all on various background mono- or combination therapies; excluding insulin)	581	18	57.5 (9.9)	3.1
Pratley (2010) [69]	1860-LIRA-DPP4	Specific GLT – GLP-1RA	Liraglutide vs sitagliptin (on background metformin only)	665	18	55.3 (9.2)	3.8
Aschner (2006) [70]	Sitagliptin Study 021	Specific GLT – DPP4i	Sitagliptin vs placebo (various background therapies; oral GLTs only)	741	18	54.2 (9.9)	6.2
Mohan (2009) [71]	-	Specific GLT – DPP4i	Sitagliptin vs placebo (various background therapies; excluding insulin)	530	18	50.9 (9.3)	10.0
Charbonnel (2006) [72]	Sitagliptin Study 020	Specific GLT – DPP4i	Sitagliptin vs placebo (both on background of metformin only)	701	18	54.5 (10.2)	6.4
Hermansen (2007) [73]	Sitagliptin Study 035	Specific GLT – DPP4i	Sitagliptin vs placebo (both groups had further groups of background glimepiride only or glimepiride and metformin)	441	18	56.0 (9.5)	3.7

First Author (year) [study ref.]	Trial Acronym	Study Category	Intervention and Comparator	Study Sample Size	Minimum Age Criterion (years)	Age of Recruited Population (years) *Mean (SD) unless otherwise specified*	Proportion Aged 18 to 39 years (%)
Rosenstock (2006) [74]	Sitagliptin Study 019	Specific GLT – DPP4i	Sitagliptin vs placebo (both on background of pioglitazone only)	353	18	56.3 (10.8)	5.4
Vilsbøll (2010) [75]	Sitagliptin Study 051	Specific GLT – DPP4i	Sitagliptin vs placebo (both on background of long- or intermediate-acting or premixed insulin with or without metformin only)	641	21	57.8 (9.2)	2.1
Dobs (2013) [76]	-	Specific GLT – DPP4i	Sitagliptin vs placebo (both on background on metformin and rosiglitazone only)	262	18	54.5 (9.0)	4.3
Goldstein (2007) [77]	Sitagliptin Study 036	Specific GLT – DPP4i	Sitagliptin and metformin dual-therapy vs sitagliptin monotherapy vs metformin monotherapy vs placebo (various background therapies; oral GLTs only)	1091	18	53.5 (9.9)	7.1
Yoon (2011) [78]	Sitagliptin Protocol 064	Specific GLT – DPP4i	Sitagliptin and pioglitazone dual-therapy vs pioglitazone monotherapy (on background of diet and exercise counselling only)	520	18	51.0 (10.7)	13.0
Aschner (2010) [79]	Sitagliptin Study 049	Specific GLT – DPP4i	Sitagliptin vs metformin (on background of diet and exercise counselling only)	894	18	56.0 (10.5)	5.3
Nauck (2007) [80]	Sitagliptin Study 024	Specific GLT – DPP4i	Sitagliptin vs glipizide (both on background of metformin only)	1172	18	56.7 (9.6)	3.3
Chan (2008) [81]	-	Specific GLT – DPP4i	Sitagliptin vs placebo followed by glipizide (on background of diet and exercise counselling only or insulin monotherapy, and all with 24hr creatinine clearance ≥30 and <50 ml/min and not on dialysis)	91	18	67.9 (9.8)	0.2

**First Author (year) [study ref.]**	**Trial Acronym**	**Study Category**	**Intervention and Comparator**	**Study Sample Size**	**Minimum Age Criterion (years)**	**Age of Recruited Population (years)** *Mean (SD) unless otherwise specified*	**Proportion Aged 18 to 39 years (%)**
Prominent studies examining the effects of diabetes self-management education and support, and intensive lifestyle interventions in adult type 2 diabetes							
Sone (2002) [82]	JDCS	DSMES	Lifestyle modification programme focussing on dietary habits, physical activity and medications adherence, delivered during standard outpatient clinics and via frequent telephone counselling vs ongoing standard care	2205	40	59.4 (7.4)	Not eligible
Young (2005) [83]	PACCTS	DSMES	Call centre support intervention with frequency of calls based on previous HbA1c measurement vs ongoing standard care	591	NR	67.0 (NR)	Not estimated (SD not reported)
Deakin (2006) [84]	X-PERT	DSMES	Face-to-face group-based education aiming to develop skills and build confidence to support informed self-management decisions vs ongoing standard care plus prearranged individual appointments with dietician, practice nurse and General Practitioner.	314	18	61.6 (10.4)	1.5
Adolfsson (2007) [85]	"Uppsala study"	DSMES	Group education focussing on empowerment and dealing with different themes of self-care in type 2 diabetes vs standard ongoing care	88	NR	63.1 (9.4)	0.5
Ko (2007) [86]	SIDEP	DSMES	Inpatient 5-day education programme with curriculum based on knowledge of type 2 diabetes, teaching of self-glucose monitoring, injection techniques, sick-day care, meal planning, physical activity, foot inspection and management of hypoglycaemia vs brief 'conventional' education	437	NR	53.7 (8.3)	3.8

**First Author (year) [study ref.]**	**Trial Acronym**	**Study Category**	**Intervention and Comparator**	**Study Sample Size**	**Minimum Age Criterion (years)**	**Age of Recruited Population (years)** *Mean (SD) unless otherwise specified*	**Proportion Aged 18 to 39 years (%)**
Davies (2008) [87]	DESMOND	DSMES	Structured group education programme delivered in the community focussing on empowerment and encouragement of participants to consider their own personal lifestyle risk factors and medication self-management vs standard ongoing care	824	18	59.5 (12.2)	4.6
Sturt (2008) [88]	Diabetes Manual	DSMES	Diabetes Manual workbook with face-to-face introduction and telephone support vs standard ongoing care	245	18	62.0 (NR)	Not estimated (SD not reported)
Gary (2009) [89]	Project Sugar 2	DSMES	Intensive multifactorial intervention using evidence-based clinical algorithms and culturally-tailored intervention action plans to address traditional and non-traditional cardiovascular risk factors vs telephone-based ‘minimal’ education	488[d]	25	58.0 (11.0)	4.2
Trento (2010) [90]	ROMEO	DSMES	Group-based education focussing on modifiable lifestyle risk factors and medication self-management through hands-on activities, group work, problem-solving, real-life simulations and role playing vs ongoing standard care	815	18	69.3 (8.1)	0.01
Walker (2011) [91]	I DO	DSMES	Regular telephone calls with health educator focussing on medications adherence and, secondarily, healthy eating and physical activity vs printed education materials	526	30	55.5 (7.3)	1.2
Ali (2016) [92]	CARRS	DSMES	Multicomponent care model including face-to-face and telephone sessions with non-physician care coordinator focussing on modifiable lifestyle risk factors, medication management, glucose self-monitoring and stress management vs ongoing standard care	1146	35	54.2 (9.2)	4.9

First Author (year) [study ref.]	Trial Acronym	Study Category	Intervention and Comparator	Study Sample Size	Minimum Age Criterion (years)	Age of Recruited Population (years) *Mean (SD) unless otherwise specified*	Proportion Aged 18 to 39 years (%)
Odnoletkova (2016) [93]	COACH	DSMES	Multifactorial tele-coaching intervention focussing on empowerment and encouragement of individuals to identify and self-manage their individuals risk factor targets vs ongoing standard care	574	18	63.1 (8.8)	3.1
Andrews (2011) [94]	Early ACTID	DSMES (diet ± physical activity only)	Intensive dietary counselling vs intensive dietary counselling plus pedometer-based activity programme vs standard ongoing care	593	30	60.0 (10.3)	2.1
Lean (2017) [95]	DiRECT	Intensive Lifestyle Intervention (Diet)	Total dietary replacement and structured support for long-term weight loss maintenance (plus withdrawal of glucose-lowering medications) vs current best-practice guidelines	298	20	54.4 (7.5)	2.0
Jenkins (2008) [96]	-	Intensive Lifestyle Intervention (Diet)	Low-glycaemic index diet vs high-cereal fibre diet	210	21	60.5 (9.4)	1.1
Davis (2009) [97]	-	Intensive Lifestyle Intervention (Diet)	Low-carbohydrate diet vs low-fat diet	105	18	53.5 (6.2)	1.0
Esposito (2009) [98]	-	Intensive Lifestyle Intervention (Diet)	Low-carbohydrate Mediterranean-style diet vs. low-fat diet	215	30	52.2 (10.9)	11.2
Krebs (2012) [99]	DEWL	Intensive Lifestyle Intervention (Diet)	Low-fat high-protein diet vs low-fat high-carbohydrate diet	419	30	58.0 (9.5)	2.3
Azadbakht (2011) [100, 101]	DASH	Intensive Lifestyle Intervention (Diet)	"Dietary Approaches to Stop Hypertension" diet vs control diet (similar to Iranian dietary composition and pattern)	31	44	55.0 (6.5)	Not eligible
van Rooijen (2004) [102]	-	Intensive Lifestyle Intervention (Supervised Exercise Training)	Supervised and home-based aerobic exercise training vs supervised relaxation sessions	149	40	54.5 (NR)	Not eligible

First Author (year) [study ref.]	Trial Acronym	Study Category	Intervention and Comparator	Study Sample Size	Minimum Age Criterion (years)	Age of Recruited Population (years) *Mean (SD) unless otherwise specified*	Proportion Aged 18 to 39 years (%)
Sigal (2007) [103]	DARE	Intensive Lifestyle Intervention (Supervised Exercise Training)	Supervised aerobic vs resistance vs combined (aerobic-plus-resistance) exercise vs non-exercise control	251	39	54.2 (7.2)	1.7
Church (2010) [104]	HART-D	Intensive Lifestyle Intervention (Supervised Exercise Training)	Supervised aerobic vs resistance vs combined (aerobic-plus-resistance) exercise training vs control (offered weekly stretching and/or relaxation sessions)	262	30	55.8 (8.7)	2.7
Balducci (2010) [105]	IDES	Intensive Lifestyle Intervention (Supervised Exercise Training)	Supervised combined (aerobic-plus-resistance) exercise training plus structured exercise counselling vs ongoing standard care	606	40	58.8 (8.6)	Not eligible
Gordon (2008) [106]	-	Intensive Lifestyle Intervention (Supervised Exercise Training)	Yoga vs "conventional" (predominantly aerobic) exercise training vs ongoing standard care	231	40	63.8 (NR)	Not eligible
Hegde (2011) [107]	-	Intensive Lifestyle Intervention (Supervised Exercise Training)	Yoga vs ongoing standard care	123	40	58.6 (9.4)	Not eligible

[a] In total, 1704 participants were recruited to UKPDS 34. 753 were randomised to the primary comparison of intensive glucose-lowering therapy with metformin vs conventional therapy. Secondary analyses compared participants in the metformin group versus individuals randomised to intensive glucose-lowering with chloropropamide, glibenclamide or insulin; [b] Median (IQR); [c] The BARI-2D study utilised a 2-by-2 factorial design; [d] >500 individuals were recruited but age data were only reported for n=488 that completed the 24-month follow-up visit.

Abbreviations: BMI: body mass index; CVD: cardiovascular disease; DPP4i: dipeptidyl peptidase 4 inhibitor; DSMES: diabetes self-management education and support; GLP-1RA: glucagon-like peptide-1 receptor agonist; GLT: glucose-lowering therapy; HbA1c: glycated haemoglobin; IQR: interquartile range; NR: not reported; SD: standard deviation; SGLT2i: sodium-glucose cotransporter 2 inhibitor; TZD: thiazolidinedione.

ESM Table 2 – Summary data for the Phase III research programmes of empagliflozin, liraglutide and sitagliptin

	Number of trials	Total number of participants	Weighted mean age (years)	Range of mean ages (years)	Range of estimated proportions of individuals aged 18 to 39 years (%)	Estimated number of individuals aged 18 to 39 years	Combined proportion of individuals aged 18 to 39 years (%)
Empagliflozin	9	6,416	57	55 to 63	0.2 to 7.3	252	3.9
Liraglutide	7	5,121	56	53 to 58	2.3 to 9.7	237	4.6
Sitagliptin	12	7,437	55	51 to 69	0.2 to 13.0	435	5.9

All trials allowed inclusion of adults aged ≥18 years, except one, which allowed those aged ≥21 years. All trials also reported age data as mean and standard deviation, thus allowing the estimation of the proportion of individuals aged 18-39 years.

ESM Table 3 – Summary data for prominent trials of diabetes self-management education and support or intensive lifestyle interventions (diet or supervised exercise training) in type 2 diabetes

	**Number of trials**	**Total number of participants**	**Weighted mean age (years)**	**Range of mean ages (years)**	**Range of estimated proportions of individuals aged 18 to 39 years (%)**	**Estimated number of individuals aged 18 to 39 years**	**Combined proportion of individuals aged 18 to 39 years (%)**
All trials reviewed							
DSMES	13	8,846	60	54 to 69	-	-	-
Diet	6	1,278	56	52 to 61	-	-	-
Supervised exercise training	6	1,622	58	54 to 64	-	-	-
Trials in which individuals aged 18 to 39 years were eligible and where the relative proportion of these individuals could be estimated							
DSMES	10	5,805	60	54 to 69	0.01 to 4.9	173	3.0
Diet	5	1,247	56	52 to 61	1.0 to 11.2	43	3.5
Supervised exercise training	2	513	55	54 to 56	1.7 to 2.7	11	2.2

ESM Figure 1 – Proportions of study populations aged between 18 and 39 years participating in cardio-renal outcomes trials

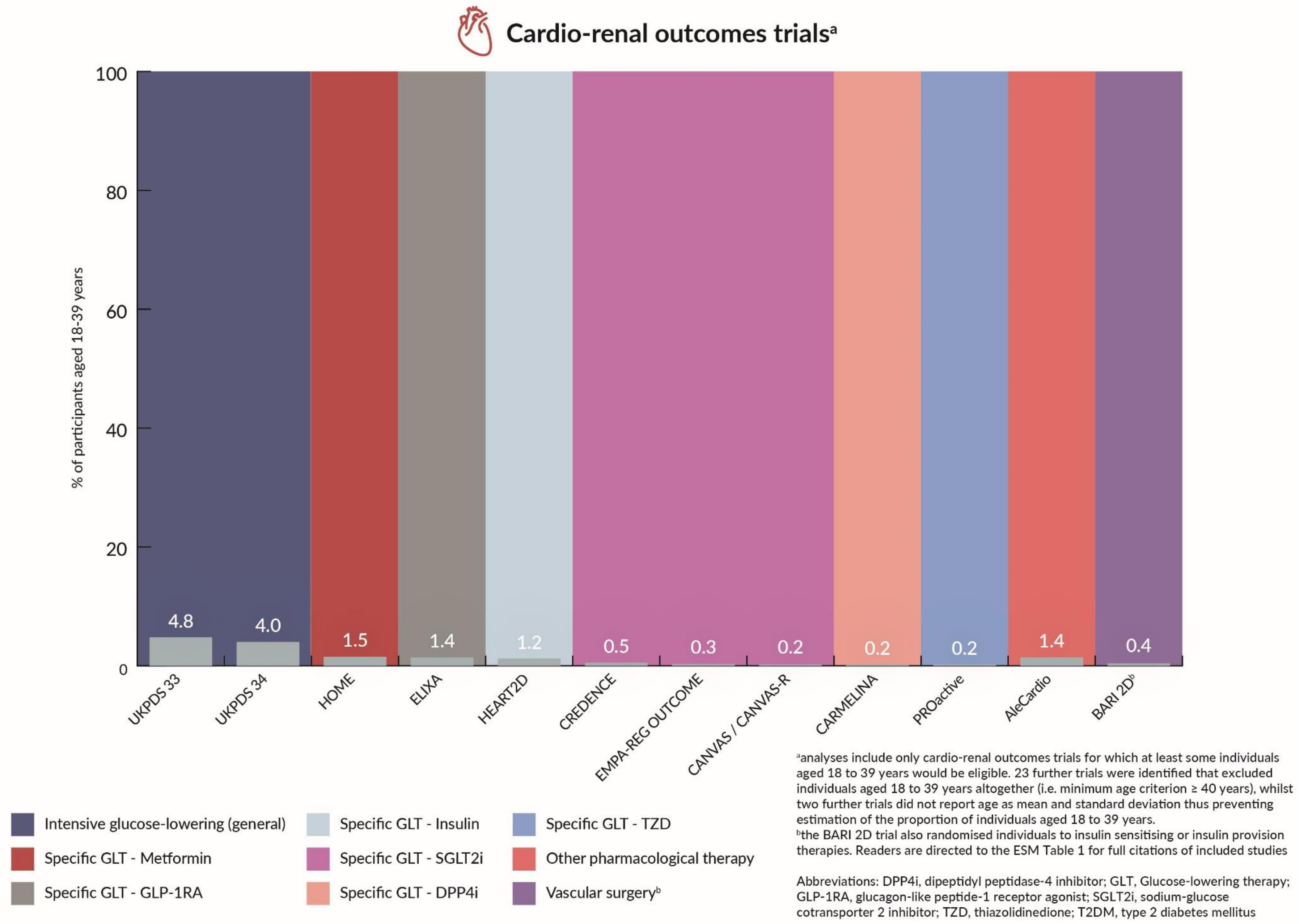

ESM Figure 2 – Proportions of study populations aged between 18 and 39 years participating in Phase III trials of pharmacological glucose-lowering therapies

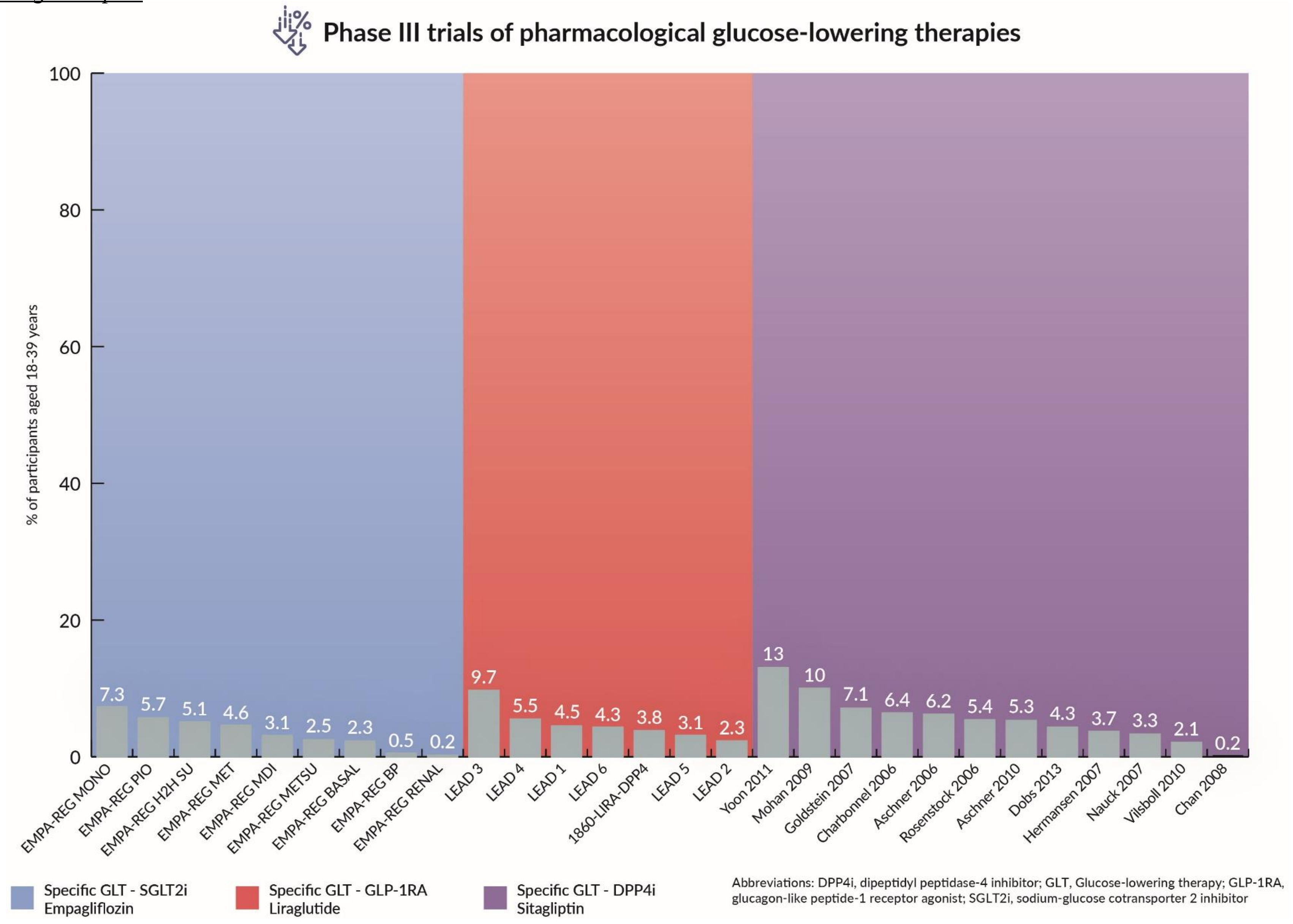

ESM Figure 3 – Proportions of study populations aged between 18 and 39 years participating in prominent trials examining the efficacy of diabetes self-management education and support or intensive lifestyle interventions in adult type 2 diabetes

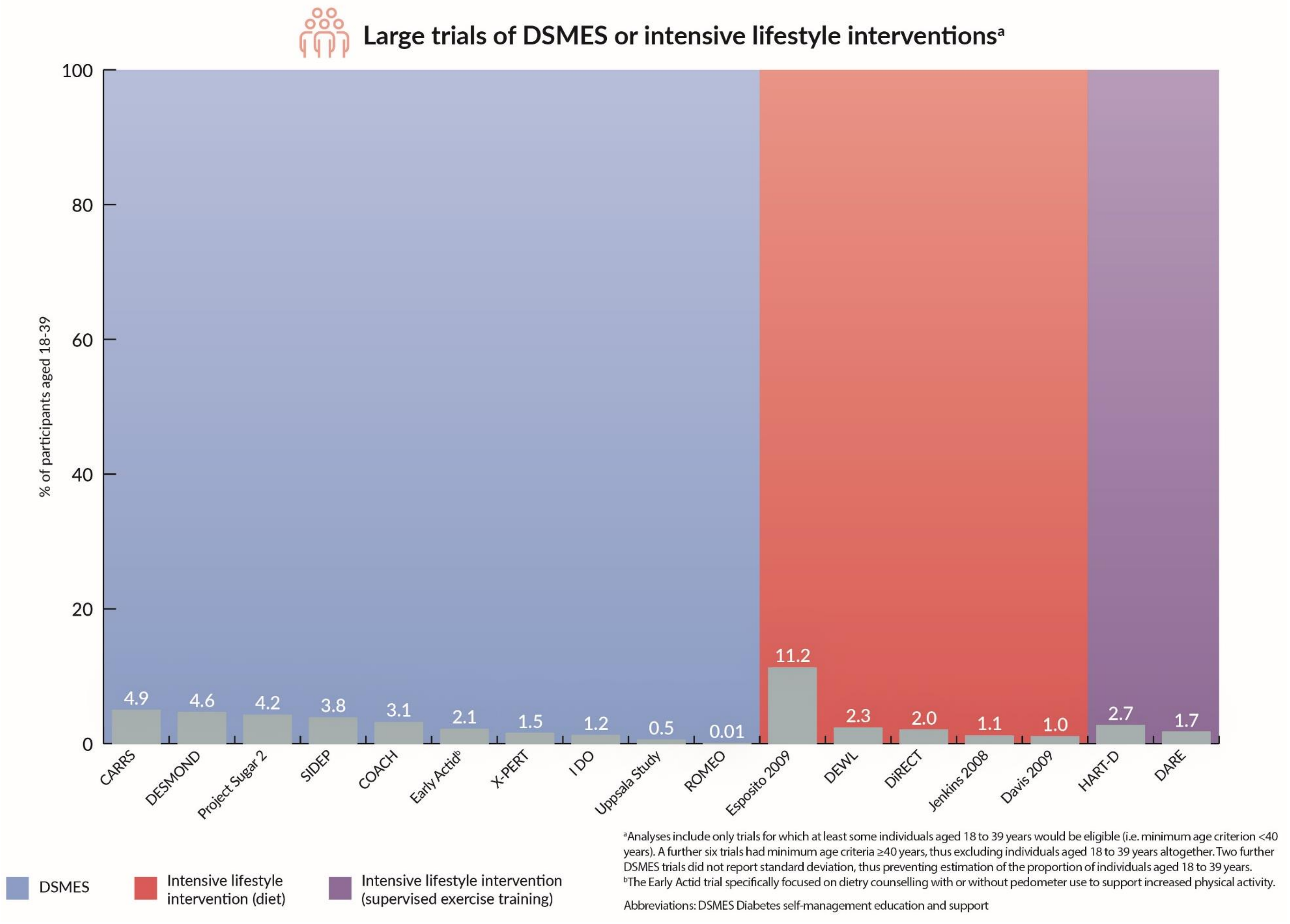

Supplementary Bibliography (including full citations of included studies)

1. Davies MJ, D'Alessio DA, Fradkin J, et al (2018) Management of hyperglycaemia in type 2 diabetes, 2018. A consensus report by the American Diabetes Association (ADA) and the European Association for the Study of Diabetes (EASD). Diabetologia 61(12):2461–2498.

2. Chatterjee S, Davies MJ, Heller S, Speight J, Snoek FJ, Khunti K (2018) Diabetes structured self-management education programmes: a narrative review and current innovations. Lancet Diabetes Endocrinol 6(2):130–142.

3. Chrvala CA, Sherr D, Lipman RD (2016) Diabetes self-management education for adults with type 2 diabetes mellitus: A systematic review of the effect on glycemic control. Patient Educ Couns 99(6):926–943.

4. He X, Li J, Wang B, et al (2017) Diabetes self-management education reduces risk of all-cause mortality in type 2 diabetes patients: a systematic review and meta-analysis. Endocrine 55(3):712–731.

5. New J, Mason J, Freemantle N, et al (2003) Specialist nurse-led intervention to treat and control hypertension and hyperlipidemia in diabetes (SPLINT): a randomized controlled trial. Diabetes Care 26:2250–2255.

6. Hanefeld M, Fischer S, Schmechel H, et al (1991) Diabetes Intervention Study: Multi-Intervention Trial in Newly Diagnosed NIDDM. Diabetes Care 14:308–317.

7. Ajala O, English P, Pinkney J (2013) Systematic review and meta-analysis of different dietary approaches. Am J Clin Nutr 97(3):505–516.

8. Sainsbury E, Kizirian NV., Partridge SR, Gill T, Colagiuri S, Gibson AA (2018) Effect of dietary carbohydrate restriction on glycemic control in adults with diabetes: A systematic review and meta-analysis. Diabetes Res Clin Pract 139:239–252.

9. O'Neil PM, Miller-Kovach K, Tuerk PW, et al (2016) Randomized controlled trial of a nationally available weight control program tailored for adults with type 2 diabetes. Obesity 24(11):2269–2277.

10. Schwingshackl L, Missbach B, Dias S, König J, Hoffmann G (2014) Impact of different training modalities on glycaemic control and blood lipids in patients with type 2 diabetes: A systematic review and network meta-analysis. Diabetologia 57(9):1789–1797.

11. Yang Z, Scott CA, Mao C, Tang J, Farmer AJ (2014) Resistance exercise versus aerobic exercise for type 2 diabetes: A systematic review and meta-analysis. Sport Med 44(4):487–499.

12. Pai LW, Li TC, Hwu YJ, Chang SC, Chen LL, Chang PY (2016) The effectiveness of regular leisure-time physical activities on long-term glycemic control in people with type 2 diabetes: A systematic review and meta-analysis. Diabetes Res Clin Pract 113:77–85.

13. Rees JL, Johnson ST, Boulé NG (2017) Aquatic exercise for adults with type 2 diabetes: a meta-analysis. Acta Diabetol 54(10):895–904.

14. Qiu S, Cai X, Schumann U, Velders M, Sun Z, Steinacker JM (2014) Impact of walking on glycemic control and other cardiovascular risk factors in type 2 diabetes: A meta-analysis. PLoS One 9(10):e109767.

15. Qiu S, Cai X, Chen X, Yang B, Sun Z (2014) Step counter use in type 2 diabetes: A meta-analysis of randomized controlled trials. BMC Med 12(1):36.

16. Higgins J, Green S (2011) Cochrance Handbook for Systematic Reviews of Interventions. John Wiley & Sons Ltd., Chichester, UK.

17. UK Prospective Diabetes Study (UKPDS) Group (1998) Effect of intensive blood-glucose control with metformin on complications in overweight patients with type 2 diabetes (UKPDS 34). Lancet 352(9131):854–865.

18. UK Prospective Diabetes Study (UKPDS) Group (1998) Intensive blood-glucose control with sulphonyulreas or insulin compared with conventional treatment and risk of complications in patients with type 2 diabetes (UKPDS 33). Lancet 352(9131):837–853.

19. Duckworth W, Abraira C, Moritz T, et al (2009) Glucose control and vascular complications in veterans with type 2 diabetes. N Engl J Med 360(2):129–139.

20. The Action to Control Cardiovascular Risk in Diabetes Study Group (2008) Effects of Intensive Glucose Lowering in Type 2 Diabetes. N Engl J Med 358(24):2545–2559.

21. The ADVANCE Collaborative Group (2008) Intensive Blood Glucose Control and Vascular Outcomes in Patients with Type 2 Diabetes. N Engl J Med 358:2560–2572.

22. Kooy A, De Jager J, Lehert P, et al (2009) Long-term effects of metformin on metabolism and microvascular and macrovascular disease in patients with type 2 diabetes mellitus. Arch Intern Med 169(6):616–625.

23. Neal B, Perkovic V, Mahaffey KW, et al (2017) Canagliflozin and cardiovascular and renal events in type 2 diabetes. N Engl J Med 377(7):644–657.

24. Perkovic V, Jardine MJ, Neal B, et al (2019) Canagliflozin and Renal Outcomes in Type 2 Diabetes and Nephropathy. N Engl J Med 380(24):2295–2306.

25. Wiviott SD, Raz I, Bonaca MP, et al (2019) Dapagliflozin and Cardiovascular Outcomes in Type 2 Diabetes. N Engl J Med 380(4):347–357.

26. Zinman B, Wanner C, Lachin JM, et al (2015) Empagliflozin, Cardiovascular Outcomes, and Mortality in Type 2 Diabetes. N Engl J Med 373(22):2117–2128.

27. Gerstein HC, Colhoun HM, Dagenais GR, et al (2019) Dulaglutide and cardiovascular outcomes in type 2 diabetes (REWIND): a double-blind , randomised placebo-controlled trial. Lancet 394(10193):121–130.

28. Hernandez AF, Green JB, Janmohamed S, et al (2018) Albiglutide and cardiovascular outcomes in patients with type 2 diabetes and cardiovascular disease (Harmony Outcomes): a double-blind, randomised placebo-controlled trial. Lancet 392(10157):1519–1529.

29. Husain M, Birkenfeld AL, Donsmark M, et al (2019) Oral Semaglutide and Cardiovascular Outcomes in Patients with Type 2 Diabetes. N Engl J Med 381:841–851.

30. Marso SP, Bain SC, Consoli A, et al (2016a) Semaglutide and Cardiovascular Outcomes in Patients with Type 2 Diabetes. N Engl J Med 375(19):1834–1844.

31. Marso SP, Daniels GH, Brown-Frandsen K, et al (2016b) Liraglutide and cardiovascular outcomes in type 2 diabetes. N Engl J Med 375(4):311–322.

32. Holman RR, Bethel MA, Mentz RJ, et al (2017) Effects of once-weekly exenatide on cardiovascular outcomes in type 2 diabetes. N Engl J Med 377(13):1228–1239.

33. Pfeffer MA, Claggett B, Diaz R, et al (2015) Lixisenatide in patients with type 2 diabetes and acute coronary syndrome. N Engl J Med 373(23):2247–2257.

34. Gantz I, Chen M, Suryawanshi S, et al (2017) A randomized, placebo-controlled study of the cardiovascular safety of the once-weekly DPP-4 inhibitor omarigliptin in patients with type 2 diabetes mellitus. Cardiovasc Diabetol 16(1):1–12.

35. Green JB, Bethel MA, Armstrong PW, et al (2015) Effect of sitagliptin on cardiovascular outcomes in type 2 diabetes. N Engl J Med 373(3):232–242.

36. Rosenstock J, Perkovic V, Johansen OE, et al (2019a) Effect of Linagliptin vs Placebo on Major Cardiovascular Events in Adults with Type 2 Diabetes and High Cardiovascular and Renal Risk: The CARMELINA Randomized Clinical Trial. JAMA 321(1):69–79.

37. Rosenstock J, Kahn SE, Johansen OE, et al (2019b) Effect of Linagliptin vs Glimepiride on Major Adverse Cardiovascular Outcomes in Patients with Type 2 Diabetes: The CAROLINA Randomized Clinical Trial. JAMA 322(12):1155–1166.

38. Scirica B, Bhatt D, Braunwald E, et al (2013) Saxagliptin and Cardiovascular Outcomes in Patients with Type 2 Diabetes Mellitus. N Engl J Med 369(14):1317–1326.

39. White WB, Cannon CP, Heller SR, et al (2013) Alogliptin after acute coronary syndrome in patients with type 2 diabetes. N Engl J Med 369(14):1327–1335.

40. Dormandy J, Charbonnel B, Eckland D, et al (2005) Secondary prevention of macrovascular events in patients with type 2 diabetes in the PROactive Study (PROspective pioglitAzone Clinical Trial In macroVascular Events): a randomised controlled trial. Lancet 366:1279–1289.

41. Yoshii H, Onuma T, Yamazaki T, et al (2014) Effects of pioglitazone on macrovascular events in patients with type 2 diabetes mellitus at high risk of stroke: The profit-J study. J Atheroscler Thromb 21(6):563–573.

42. Vaccaro O, Masulli M, Nicolucci A, et al (2017) Effects on the incidence of cardiovascular events of the addition of pioglitazone versus sulfonylureas in patients with type 2 diabetes inadequately controlled with metformin (TOSCA.IT): a randomised, multicentre trial. Lancet Diabetes Endocrinol 5(11):887–897.

43. Home PD, Pocock SJ, Beck-Nielsen H, et al (2009) Rosiglitazone evaluated for cardiovascular outcomes in oral agent combination therapy for type 2 diabetes (RECORD): a multicentre, randomised, open-label trial. Lancet 373(9681):2125–2135.

44. Gerstein HC, Bosch J, Dagenais GR, et al (2012) Basal insulin and cardiovascular and other outcomes in dysglycemia. N Engl J Med 367(4):319–328.

45. Marso SP, McGuire DK, Zinman B, et al (2017) Efficacy and safety of degludec versus Glargine in Type 2 Diabetes. N Engl J Med 377(8):723–732.

46. Raz I, Jermendy G, Wilson PWF, et al (2009) Effects of prandial versus fasting glycemia on cardiovascular outcomes in type 2 diabetes: The HEART2D trial. Diabetes Care 32(3):381–386.

47. Lincoff AM, Tardif JC, Schwartz GG, et al (2014) Effect of aleglitazar on cardiovascular outcomes after acute coronary syndrome in patients with type 2 diabetes mellitus: The AleCardio randomized clinical trial. JAMA 311(15):1515–1525.

48. Wing R, Group LAR (2013) Cardiovascular Effects of Intensive Lifestyle Intervention in Type 2 Diabetes. N Engl J Med 369(2):145–154.

49. The BARI 2D Study Group (2009) A randomized trial of therapies for type 2 diabetes and coronary artery disease. N Engl J Med 360(24):2503–2515.

50. Gaede P, Vedel P, Larsen N, Jensen GV, Parving H-H, Pedersen O (2003) Multifactorial intervention and cardiovascular disease in patients with type 2 diabetes. N Engl J Med 348(5):383–393.

51. Hansen LJ, Siersma V, Beck-Nielsen H, De Fine Olivarius N (2013) Structured personal care of type 2 diabetes: A 19 year follow-up of the study Diabetes Care in General Practice (DCGP). Diabetologia 56(6):1243–1253.

52. Ueki K, Sasako T, Okazaki Y, et al (2017) Effect of an intensified multifactorial intervention on cardiovascular outcomes and mortality in type 2 diabetes (J-DOIT3): an open-label, randomised controlled trial. Lancet Diabetes Endocrinol 5(12):951–964.

53. Cannon CP, Mcguire DK, Pratley R, et al (2018) Design and baseline characteristics of the eValuation of ERTugliflozin effIcacy and Safety CardioVascular outcomes trial (VERTIS-CV). Am Heart J 206:11–23.

54. Roden M, Weng J, Eilbracht J, et al (2013) Empagliflozin monotherapy with sitagliptin as an active comparator in patients with type 2 diabetes: A randomised, double-blind, placebo-controlled, phase 3 trial. Lancet Diabetes Endocrinol 1(3):208–219.

55. Häring HU, Merker L, Seewaldt-Becker E, et al (2014) Empagliflozin as add-on to metformin in patients with type 2 diabetes: A 24-week, randomized, double-blind, placebo-controlled trial. Diabetes Care 37(6):1650–1659.

56. Häring HU, Merker L, Seewaldt-Becker E, et al (2013) Empagliflozin As Add-on to Metformin Plus Sulfonylurea in Patients With Type 2 Diabetes: A 24-week, randomzed, double-blinded, placebo-controlled trial. Diabetes Care 36:3396–3404.

57. Kovacs C, Seshiah V, Swallow R, et al (2014) Empagliflozin improves glycaemic and weight control as add-on therapy to pioglitazone or pioglitazone plus metformin in patients with type 2 diabetes: a 24-week, randomized, placebo-controlled trial. Diabetes, Obes Metab 16:147–158.

58. Rosenstock J, Jelaska A, Zeller C, Kim G, Broedl UC, Woerle HJ (2015) Impact of empagliflozin added on to basal insulin in type 2 diabetes inadequately controlled on basal insulin: A 78-week randomized, double-blind, placebo-controlled trial. Diabetes, Obes Metab 17(10):936–948.

59. Rosenstock J, Jelaska A, Frappin G, et al (2014) Improved glucose control with weight loss, lower insulin doses, and no increased hypoglycemia with empagliflozin added to titrated multiple daily injections of insulin in obese inadequately controlled type 2 diabetes. Diabetes Care 37(7):1815–1823.

60. Ridderstråle M, Andersen KR, Zeller C, Kim G, Woerle HJ, Broedl UC (2014) Comparison of empagliflozin and glimepiride as add-on to metformin in patients with type 2 diabetes: A 104-week randomised, active-controlled, double-blind, phase 3 trial. Lancet Diabetes Endocrinol 2(9):691–700.

61. Tikkanen I, Narko K, Zeller C, et al (2015) Empagliflozin reduces blood pressure in patients with type 2 diabetes and hypertension. Diabetes Care 38(3):420–428.

62. Barnett AH, Mithal A, Manassie J, et al (2014) Efficacy and safety of empagliflozin added to existing antidiabetes treatment in patients with type 2 diabetes and chronic kidney disease: A randomised, double-blind, placebo-controlled trial. Lancet Diabetes Endocrinol 2(5):369–384.

63. Marre M, Shaw J, Brändle M, et al (2009) Liraglutide, a once-daily human GLP-1 analogue, added to a sulphonylurea over 26 weeks produces greater improvements in glycaemic and weight control compared with adding rosiglitazone or placebo in subjects with Type 2 diabetes (LEAD-1 SU). Diabet Med 26(3):268–278.

64. Nauck M, Frid A, Hermansen K, et al (2009) Efficacy and Safety Comparison of Liraglutide,Glimepiride, and Placebo, All in Combination with Metformin, in Type 2 Diabetes. Diabetes Care 32:84–90.

65. Garber A, Henry R, Ratner R, et al (2009) Liraglutide versus glimepiride monotherapy for type 2 diabetes (LEAD-3 Mono): a randomised, 52-week, phase III, double-blind, parallel-treatment trial. Lancet 373(9662):473–481.

66. Zinman B, Gerich J, Buse J, et al (2009) Efficacy and safety of the human GLP-1 analog liraglutide in combination with metformin and TZD in patients with type 2 diabetes mellitus (LEAD-4 Met+TZD). Diabetes Care 32(7):1224–1230.

67. Buse JB, Rosenstock J, Sesti G, et al (2009) Liraglutide once a day versus exenatide twice a day for type 2 diabetes: a 26-week randomised, parallel-group, multinational, open-label trial (LEAD-6). Lancet 374(9683):39–47.

68. Russell-Jones D, Vaag A, Schmitz O, et al (2009) Liraglutide vs insulin glargine and placebo in combination with metformin and sulfonylurea therapy in type 2 diabetes mellitus (LEAD-5 met+SU): A randomised controlled trial. Diabetologia 52(10):2046–2055.

69. Pratley RE, Nauck M, Bailey T, et al (2010) Liraglutide versus sitagliptin for patients with type 2 diabetes who did not have adequate glycaemic control with metformin: A 26-week, randomised, parallel-group, open-label trial. Lancet 375(9724):1447–1456.

70. Aschner P, Kipnes MS, Lunceford JK, Sanchez M, Mickel C, Williams-Herman DE (2006) Effect of the dipeptidyl peptidase-4 inhibitor sitagliptin as monotherapy on glycemic control in patients with type 2 diabetes. Diabetes Care 29(12):2632–2637.

71. Mohan V, Yang W, Son HY, et al (2009) Efficacy and safety of sitagliptin in the treatment of patients with type 2 diabetes in China, India, and Korea. Diabetes Res Clin Pract 83(1):106–116.

72. Charbonnel B, Karasik A, Liu J, Wu M, Meininger G (2006) Efficacy and safety of the dipeptidyl peptidase-4 inhibitor sitagliptin added to ongoing metformin therapy in patients with type 2 diabetes inadequately controlled with metformin alone. Diabetes Care 29(12):2638–2643.

73. Hermansen K, Kipnes M, Luo E, Fanurik D, Khatami H, Stein P (2007) Efficacy and safety of the dipeptidyl peptidase-4 inhibitor, sitagliptin, in patients with type 2 diabetes mellitus inadequately controlled on glimepiride alone or on glimepiride and metformin. Diabetes, Obes Metab 9(5):733–745.

74. Rosenstock J, Brazg R, Andryuk PJ, et al (2006) Efficacy and Safety of the Dipeptidyl Peptidase-4 Inhibitor Sitagliptin Added to Ongoing Pioglitazone Therapy in Patients with Type 2 Diabetes: A 24-week, multicenter, randomized, double-blind, placebo-controlled, parallel-group study. Clin Ther 28(12):1556–1568.

75. Vilsbøll T, Rosenstock J, Yki-Järvinen H, et al (2010) Efficacy and safety of sitagliptin when added to insulin therapy in patients with type 2 diabetes. Diabetes, Obes Metab 12(2):167–177.

76. Dobs AS, Goldstein BJ, Aschner P, et al (2013) Efficacy and safety of sitagliptin added to ongoing metformin and rosiglitazone combination therapy in a randomized placebo-controlled 54-week trial in patients with type 2 diabetes. J Diabetes 5(1):68–79.

77. Goldstein BJ, Feinglos MN, Lunceford JK, Johnson J, Williams-Herman DE (2007) Effect of Initial Combination Therapy With Sitagliptin, a Dipeptidyl Peptidase-4 Inhibitor, and Metformin on Glycemic Control in Patients with Type 2 Diabetes. Diabetes Care 30(8):1979–1987.

78. Yoon KH, Shockey GR, Teng R, et al (2011) Effect of initial combination therapy with sitagliptin, a dipeptidyl peptidase-4 inhibitor, and pioglitazone on glycemic control and measures of β-cell function in patients with type 2 diabetes. Int J Clin Pract 65(2):154–164.

79. Aschner P, Katzeff HL, Guo H, et al (2010) Efficacy and safety of monotherapy of sitagliptin compared with metformin in patients with type 2 diabetes. Diabetes, Obes Metab 12(3):252–261.

80. Nauck MA, Meininger G, Sheng D, et al (2007) Efficacy and safety of the dipeptidyl peptidase-4 inhibitor, sitagliptin, compared with the sulfonylurea, glipizide, in patients with type 2 diabetes inadequately controlled on metformin alone: A randomized, double-blind, non-inferiority trial. Diabetes, Obes Metab 9(2):194–205.

81. Chan JCN, Scott R, Arjona Ferreira JC, et al (2008) Safety and efficacy of sitagliptin in patients with type 2 diabetes and chronic renal insufficiency. Diabetes, Obes Metab 10(7):545–555.

82. Sone H, Katagiri A, Ishibashi S, et al (2002) Effects of lifestyle modifications on patients with type 2 diabetes: The Japan Diabetes Complications Study (JDCS) study design, baseline analysis and three year-interim report. Horm Metab Res 34(9):509–515.

83. Young R, Taylor J, Friede T, et al (2005) Pro-Active Call Center Treatment Support (PACCTS) to Improve Glucose Control in Type 2 Diabetes A randomized controlled trial. Diabetes Care 28:278–282.

84. Deakin TA, Cade JE, Williams R, Greenwood DC (2006) Structured patient education: The Diabetes X-PERT Programme makes a difference. Diabet Med 23(9):944–954.

85. Adolfsson ET, Walker-Engström ML, Smide B, Wikblad K (2007) Patient education in type 2 diabetes-A randomized controlled 1-year follow-up study. Diabetes Res Clin Pract 76(3):341–350.

86. Ko SH, Song KH, Kim SR, et al (2007) Long-term effects of a structured intensive diabetes education programme (SIDEP) in patients with Type 2 diabetes mellitus - A 4-year follow-up study. Diabet Med 24(1):55–62.

87. Davies MJ, Heller S, Skinner TC, et al (2008) Effectiveness of the diabetes education and self management for ongoing and newly diagnosed (DESMOND) programme for people with newly diagnosed type 2 diabetes: Cluster randomised controlled trial. BMJ 336(7642):491–495.

88. Sturt JA, Whitlock S, Fox C, et al (2008) Effects of the Diabetes Manual 1:1 structured education in primary care. Diabet Med 25(6):722–731.

89. Gary T, Batts-Turner M, Yeh H-C, et al (2009) The effects of a nurse case manager and a community health worker team on diabetic control, emergency department visits, and hospitalizations among urban African Americans with type 2 diabetes mellitus. A randomized controlled trial. Arch Intern Med 169(19):1788–1794.

90. Trento M, Gamba S, Gentile L, et al (2010) Rethink Organization to iMprove Education and Outcomes (ROMEO): A multicenter randomized trial of lifestyle intervention by group care to manage type 2 diabetes. Diabetes Care 33(4):745–747.

91. Walker EA, Shmukler C, Ullman R, Blanco E, Scollan-Koliopoulus M, Cohen HW (2011) Results of a successful telephonic intervention to improve diabetes control in urban adults: A randomized trial. Diabetes Care 34(1):2–7.

92. Ali MK, Singh K, Kondal D, et al (2016) Effectiveness of a multicomponent quality improvement strategy to improve achievement of diabetes care goals a randomized, controlled trial. Ann Intern Med 165(6):399–408.

93. Odnoletkova I, Goderis G, Nobels F, et al (2016) Optimizing diabetes control in people with Type 2 diabetes through nurse-led telecoaching. Diabet Med 33(6):777–785.

94. Andrews R, Cooper AR, Montgomery AA, et al (2011) Diet or diet plus physical activity versus usual care in patients with newly diagnosed type 2 diabetes: The Early ACTID randomised controlled trial. Lancet 378(9786):129–139.

95. Lean MEJ, Leslie WS, Barnes AC, et al (2018) Primary care-led weight management for remission of type 2 diabetes (DiRECT): An open-label, cluster-randomised trial. Lancet 391(10120):541–551.

96. Jenkins DJA, Kendall CWC, McKeown-Eyssen G, et al (2008) Effect of a low-glycemic index or a high-cereal fiber diet on type 2 diabetes: A randomized trial. JAMA 300(23):2742–2753.

97. Davis N, Tomuta N, Schechter C, et al (2009) Comparative Study of the Effects of a 1-Year Dietary Intervention of a Low-Carbohydrate Diet Versus a Low-Fat Diet on Weight and Glycemic Control in Type 2 Diabetes. Diabetes Care 32:1147–1152.

98. Esposito K, Maiorino MI, Ciotola M, et al (2009) Effects of a mediterranean-style diet on the need for antihyperglycemic drug therapy in patients with newly diagnosed type 2 diabetes: A randomized trial. Ann Intern Med 151:306–314.

99. Krebs JD, Elley CR, Parry-Strong A, et al (2012) The Diabetes Excess Weight Loss (DEWL) Trial: A randomised controlled trial of high-protein versus high-carbohydrate diets over 2 years in type 2 diabetes. Diabetologia 55(4):905–914.

100. Azadbakht L, Fard NRP, Karimi M, et al (2011) Effects of the Dietary Approaches to Stop Hypertension (DASH) eating plan on cardiovascular risks among type 2 diabetic patients: A randomized crossover clinical trial. Diabetes Care 34(1):55–57.

101. Azadbakht L, Surkan P, Esmaillzadeh A, Willett WC (2011) The Dietary Approaches to Stop Hypertension Eating Plan Affects C-Reactive Protein, Coagulation Abnormalities, and Hepatic Function Tests among Type 2 Diabetic Patients. J Nutr 141:1083–1088.

102. van Rooijen AJ, Rheeder P, Eales CJ, Becker PJ (2004) Effect of exercise versus relaxation of haemoglobin A1C in black females with type 2 diabetes mellitus. QJM 97(6):343–351.

103. Sigal RJ, Kenny GP, Boulé NG, et al (2007) Effects of aerobic training, resistance training, or both on glycemic control in type 2 diabetes: A randomized trial. Ann Intern Med 147(6):357–369.

104. Church TS, Blair SN, Cocreham S, et al (2010) Effects of aerobic and resistance training on hemoglobin A1c levels in patients with type 2 diabetes: A randomized controlled trial. JAMA 304(20):2253–2262.

105. Balducci S, Zanuso S, Nicolucci A, et al (2010) Effect of an Intensive Exercise Intervention Strategy on Modifiable Cardiovascular Risk Factors in Subjects With Type 2 Diabetes Mellitus. Ann Intern Med 170(20):1794–1803.

106. Gordon L, Morrison EY, McGrowder DA, et al (2008) Changes in clinical and metabolic parameters after exercise therapy in patents with type 2 diabetes. Arch Med Sci 4(4):427–437.

107. Hegde S V., Adhikari P, Kotian S, Pinto VJ, D'Souza S, D'Souza V (2011) Effect of 3-month yoga on oxidative stress in type 2 diabetes with or without complications: A controlled clinical trial. Diabetes Care 34(10):2208–2210.
